# Serum Vitamins D, B9 and B12 in Greek Patients with Inflammatory Bowel Diseases

**DOI:** 10.3390/nu12123734

**Published:** 2020-12-04

**Authors:** Aristea Gioxari, Charalampia Amerikanou, Efstathia Papada, Evangelia Zioga, Andreas D. Georgoulis, George Bamias, Andriana C. Kaliora

**Affiliations:** 1 Department of Dietetics and Nutritional Science, School of Health Science and Education, Harokopio University, 70 El. Venizelou Ave., 17671 Athens, Greece; arisgiox@gmail.com (A.G.); amerikanou@windowslive.com (C.A.); efpapada@gmail.com (E.P.); evazioga92@hotmail.com (E.Z.); antreas.geo94@gmail.com (A.D.G.); 2 GI Unit, 3rd Academic Department of Internal Medicine, National and Kapodistrian University of Athens, Sotiria Hospital, 115 27 Athens, Greece; gbamias@gmail.com

**Keywords:** IBD, vitamin D, folate, cobalamin, inflammation, oxidative stress

## Abstract

Deficiencies in vitamin D, folate and cobalamin are common in Inflammatory Bowel Disease (IBD). The aim of the present study was to assess serum levels of these vitamins in IBD adults based on the respective serum cut off values for vitamin deficiencies, and to explore possible associations with IBD-related biomarkers and nutritional intake. A cross-sectional study was carried out and patients with Crohn’s disease (CD) or ulcerative colitis (UC) from Attica-Greece were enrolled. Medical and dietary history, clinical examination and blood/stool biomarkers were evaluated. In total, 87 patients participated in the study. Serum levels of 25(OH)D, folate and cobalamin were deficient in 36.8%, 18.4% and 5.7% of patients, respectively. Linear regression analysis in the overall patients showed positive associations between (a) serum 25(OH)D with serum iron (beta = 0.083, *p* = 0.005) and (b) serum cobalamin with total bilirubin (beta = 0.357, *p* = 0.020) and direct bilirubin (beta = 0.727, *p* = 0.033), adjusting for age, sex, body mass index (BMI), disease activity and duration, smoking, nutritional intake and season of recruitment. In CD patients (*N* = 54), a negative linear association between serum folate and fecal lysozyme was evident (beta = −0.009, *p* = 0.020). No associations were found for UC patients (*N* = 33). The serum vitamin profile may be a complementary biomarker for the evaluation of disease activity next to serum and stool inflammatory biomarkers.

## 1. Introduction

Inflammatory Bowel Disease (IBD) refers mainly to Crohn’s disease (CD) and ulcerative colitis (UC), which are characterised by chronic relapsing of intestinal inflammation. Despite several similarities, the two entities differ in genetic predispositions, inflammatory response, as well as in clinical, endoscopic and histological characteristics [1].

IBD has been characterised as a global disease with estimated prevalence exceeding 0.3% in North America, Oceania, and many countries in Europe [2]. Disease pathogenesis is still unknown; however, it involves a persistent imbalance between pro-inflammatory and anti-inflammatory signalling in response to environmental triggers in genetically predisposed individuals [3]. Defects in the mucosal epithelial barrier function allow exposure to gut luminal antigens, leading to stimulation of dendritic cells and ongoing activation of the inflammatory cascade [4]. In the presence of inflammation, reactive oxygen species inhibit antioxidant mechanisms that result in lipid peroxidation and oxidative stress [5].

IBD has a negative impact on quality of life [6], while malnutrition is very common in patients during remission and relapse. Malnutrition is correlated with prolonged hospitalisation, peri-operative complications and high mortality rates [7]. Decreased dietary intake, nutrient malabsorption, and increased energy expenditure, which is not matched with increased intake, are responsible for macro- and micronutrient deficiencies, weight loss, deficits in body composition, and loss of muscle strength [8].

Among the most frequent micronutrient deficiencies in IBD patients are those in vitamin D, folate (vitamin B9) and cobalamin (vitamin B12) [7]. In healthy populations, cut off serum values of 20 ng/mL, 3 ng/mL, and 200 pg/mL have been defined for diagnosis of deficiencies of vitamins D, B9 and B12, respectively [9,10]. Deficiency of these vitamins may lead to impairments of clinical, biochemical and inflammatory status in IBD patients. For instance, vitamin B9 and B12 deficiencies have been associated with anaemia in IBD [11]. Vitamin B9 is essential in the biological methylation and synthesis of nucleotides [11], while its role in the immunoregulation has been well-recognised [12]. Together with vitamin B12 deficiency, B9 deficiency inhibits the conversion of the pro-oxidant and the pro-inflammatory homocysteine to methionine, resulting in homocysteine accumulation in the blood and intestinal mucosa of IBD patients [13]. Absorption of vitamin B9 takes place in the jejunum, while vitamin B12 requires activation by the intrinsic factor before its absorption in the terminal ileum [14,15]. Consequently, vitamin B12 deficiency is common in CD patients in whom the ileum is affected [7]. Deficiency of vitamin B9 may be attributed to the reduced intake of foods containing the vitamin, but recent studies suggest that the use of methotrexate or sulfasalazine for IBD treatment can induce the vitamin’s malabsorption [7]. Vitamin D deficiency is very common in IBD patients and there is growing evidence that vitamin D has a protective role in regulating gut inflammation and the microbiome [16,17].

The aim of the present study was to assess the status of vitamins D, B9 and B12 in adult patients with IBD, based on the respective serum threshold values for vitamin deficiencies [9,10]. Additionally, our goal was to determine the associations between vitamin levels and clinical, biochemical, serum and stool inflammatory indices, markers of oxidative damage/antioxidant capacity and nutritional parameters.

## 2. Materials and Methods

### 2.1. Ethics

Study protocol was reviewed and approved by the Ethics Committee of Harokopio University (49/29-10-2015). The study was performed according to the principles of Helsinki Declaration and was in line with terms of Good Clinical Practice. All staff were properly trained and laboratory techniques were standardised.

### 2.2. Study Design

The present work is a cross-sectional study. Adult male and female outpatients with endoscopically confirmed Crohn’s Disease or Ulcerative Colitis from the region of Attica-Greece were enrolled through an announcement to the Hellenic Society of Crohn’s Disease and Ulcerative Colitis Patients. Recruitment took place between May 2016 and June 2017.

Patients who agreed to participate in the study were provided with a detailed information leaflet describing the aims, methods, benefits and potential hazards of the study. A written informed consent was provided by each recruited patient, and a copy of the signed consent was kept by the patient. Patients were excluded from the study if they were <18 years, and/or had one of the following: positive stool culture for enteric pathogens or Clostridium difficile toxin; bowel surgery performed ≤ 3 months prior to screening; short bowel syndrome; intra-abdominal abscess or fistula with clinical or radiological evidence of an associated abscess; stoma surgery; enteral or parenteral nutrition; alcohol or drug abuse; vitamin or inorganic supplement use ≤ 6 months prior to screening; a vegan or macrobiotic diet ≤ 5 years prior to screening; any malignancy ≤ 1 year or cancer survivors ≤ 10 years prior to screening; cardiovascular event with subsequent hospitalisation; peptic ulcer; pregnant or lactating.

### 2.3. Assessment

#### 2.3.1. Medical History

A detailed medical history of each patient was obtained by a gastroenterologist, who recorded both IBD information (i.e., IBD initiation, disease location, age at diagnosis, complications, and treatment) and general data (i.e., allergic reactions and smoking habits). The Partial Mayo Score (PMS) and the Harvey–Bradshaw index (HBI) were used to evaluate the disease activity in patients with UC and CD, respectively [18,19].

#### 2.3.2. Quality of Life Assessment

The Inflammatory Bowel Disease Questionnaire (IBDQ) was used to assess quality of life. The IBDQ consists of 32 questions about bowel, social, systemic, and emotional performance. Scoring ranges between 32 to 224 points, and a higher score indicates a better quality of life [20].

#### 2.3.3. Blood Sample Collection

After overnight fasting, standard blood withdrawal (20 mL) was performed for each participant (10 mL for serum and 10 mL for plasma isolation). For plasma isolation, EDTA was used as an anticoagulant. To collect serum, whole blood was previously allowed to clot at room temperature for 20 min. Whole blood samples were centrifuged at 3000 rpm for 10 min at 4 °C. Serum and plasma samples were then aliquoted and stored at −80 °C until further analysis for inflammatory and oxidative stress biomarkers. For routine biochemical analysis, freshly drawn samples were used.

#### 2.3.4. Stool Sampling

All enrolled patients provided stool samples with an easy-to-use stool preparation system, which comprised a tube filled with extraction buffer IDK Extract^®^ and a sampling stick (Immundiagnostik, AG, Bensheim, Germany). After sampling, the sampling stick was pushed through a collar that striped away the excess stool retaining 15 mg of the sample, which finally entered the buffer containing tube. Stool extracts were kept for a maximum of 9 days at −20 °C until further analysis.

#### 2.3.5. Anthropometric Measurements

Body weight was measured to the nearest 0.1 kg. Height was measured with a standard stadiometer to the nearest millimetre. Both measurements were performed twice. Body mass index, calculated as the ratio of average weight (kg) to the square of average height (m^2^), was assessed according to the WHO|Global Database on Body Mass Index (BMI) [21].

#### 2.3.6. Dietary Intake

Dietary intake was assessed by an experienced dietitian using the 24-h recall record. Diet composition in regards to vitamins D, B12 and B9 was assessed with the software package Nutritionist Pro™ (Axxya Systems, Stafford, TX, USA). Fortification of foods with vitamins D, B9 and B12 is not mandatory in Greece. In order to assess consumption of commercially available fortified food products, nutritional labels on food packaging were recorded and registered in the software package. In total, three 24 h-recall records of non-consecutive days were collected for each participant within a frame of two weeks.

#### 2.3.7. Biochemical Analyses

Serum iron (Fe), glucose (GLU), total cholesterol (TC), high-density lipoprotein (HDL), low-density lipoprotein (LDL), triglycerides (TG) and plasma fibrinogen were quantified with an automatic biochemical analyser using the manufacturer’s reagents (Cobas 8000 analyser, Roche Diagnostics GmbH, Mannheim, Germany). Routine laboratory tests also included serum amylase, albumin (ALB), urea, uric acid (UA), total bilirubin (TBIL), direct bilirubin (DBIL), lactate dehydrogenase (LDH), aspartate aminotransferase (AST), alanine aminotransferase (ALT), γ-glutamyl transferase (γ-GT), alkaline phosphatase (ALP). C-reactive protein (CRP) in serum was measured by turbidimetry (Cobas 8000 analyser, Roche Diagnostics GmbH, Mannheim, Germany).

An automated chemiluminescence system was used (Cobas e 801 analytical module, Roche Diagnostics GmbH, Mannheim, Germany) to determine the vitamin concentrations in serum. Deficiency of vitamin D was defined as circulating 25(OH)D < 20 ng/mL, according to the Endocrine Society Guidelines [9]. Serum deficiencies of vitamins B9 and B12 were defined as <3 ng/mL, and <200 pg/mL, respectively [10].

To assess inflammatory status, serum levels of interleukin (IL)-6 (R&D Systems, Inc., Minneapolis, MN, USA), IL-10 (OriGene Technologies, Inc., Rockville, MD, USA), IL-11 (Boster Biological Technology, Pleasanton, CA, USA), IL-17 (Boster Biological Technology, Pleasanton, CA, USA), and IL-22 (Boster Biological Technology, Pleasanton, CA, USA), were measured using sandwich ELISA. Levels of calprotectin, lysozyme, and lactoferrin were quantified in stool samples using sandwich ELISA (Immundiagnostik, AG, Bensheim, Germany).

Oxidative stress was evaluated with the following tests. To assess serum antioxidant capacity, the resistance of serum lipids to copper sulphate-induced oxidisability (expressed as lag time) [22] was measured using a Biotek PowerWave XS2 ELISA reader. Serum myeloperoxidase (MPO) activity was measured using a colorimetric assay kit according to manufacturer’s instructions (Thermo Fisher Scientific Inc., Waltham, MA, USA). Serum UA and ALB quantified with an automatic biochemical analyser as described above, as well as plasma cysteine, were also used as oxidative stress markers [23,24,25]. Plasma levels of free cysteine were evaluated applying a Gas Chromatography Mass Spectrometry (GC-MS) method as has been previously described [26].

### 2.4. Statistical Analysis

Data are expressed as mean ± standard deviation (SD). The Kolmogorov–Smirnov test was used to assess normality of the distribution. The differences between two independent groups were analysed using Student’s t-test for normally distributed variables or Mann–Whitney U test for those not normally distributed. Spearman’s correlation tests were used for the correlation analysis. For parameters showing a significant bivariate correlation with serum vitamin levels, linear regression models were applied, with serum vitamin levels being log transformed where necessary.

Regression models were fit to analyse the relationship of serum vitamins with disease-related markers, adjusted for age, sex and BMI (model 1), or age, sex, BMI, disease, activity, disease duration, smoking, nutritional intake and sun exposure (model 2) as covariates. Depending on the season of recruitment and blood collection, we categorized patients to those recruited during winter and spring and those recruited during summer and autumn when the average sunshine duration in Greece is >250 h monthly on average.

## 3. Results

### 3.1. Analyses in Overall IBD Population

Eighty-seven IBD patients (*N* = 87) met our criteria and were enrolled in the study. All participants were adults with mean age 39.6 (±13.1) years. Demographic and clinical characteristics of enrolled patients are shown in Table 1. As presented in Table 2, overall, 32 patients were detected with vitamin D deficiency, 16 patients with vitamin B9 deficiency and only five patients with vitamin B12 deficiency. In the case of vitamin B12, the low prevalence of vitamin B12 deficiency (5.7%) did not allow for further statistical analysis. The biochemical, stool inflammatory, as well as inflammatory and oxidative stress profile in serum or plasma based on vitamin status are shown in Supplementary Tables S1–S3, respectively (Supplementary Material).

#### 3.1.1. Serum 25(OH)D

In the overall study population, mean serum 25(OH)D was 20.7 ± 7.5 ng/mL (Table 2), with mean concentrations of 25.5 ± 5.2 and 14.2 ± 3.1 ng/mL for vitamin D non-deficient and deficient patients, respectively (*p* < 0.001). HBI score did not differ between the vitamin D deficient and the non-deficient patients (4.3 ± 3.2 vs. 4.1 ± 2.8 respectively, *p* = 0.79), however correlation analysis revealed that HBI was negatively correlated with serum 25(OH)D in all study participants (*r* = −0.481, *p* = 0.001). No difference in IBDQ score was observed between the vitamin D deficient and the non-deficient participants (164.3 ± 28.7 vs. 159.7 ± 32.5 respectively, *p* = 0.67). Additionally, serum Fe differed significantly between the two vitamin D subgroups, with higher levels found in the non-deficient patients versus the deficient subgroup (68.2 ± 30.4 vs. 53.7 ± 30.6 μg/dL, *p* = 0.048) (Table S1). Positive correlations of serum 25(OH)D with serum Fe (*r* = 0.303, *p* = 0.010) and dietary folate equivalent (DFE) intake (*r* = 0.307, *p* = 0.009) were detected.

#### 3.1.2. Serum Vitamin B9

In the overall study population, mean serum vitamin B9 was 5.3 ± 2.7 ng/mL (Table 2), with mean concentrations of 6.3 ± 2.4 and 2.5 ± 0.4 ng/mL in vitamin B9 non-deficient and deficient patients, respectively (*p* < 0.001). Significantly lower HBI and significantly higher IBDQ scores were observed in patients with serum vitamin B9 ≥ 3 ng/mL compared to those with vitamin B9 deficiency (HBI: 3.4 ± 2.5 vs. 6.6 ± 3.1, respectively, *p* = 0.003 and IBDQ: 166.5 ± 29.7 vs. 143.5 ± 29.3, respectively, *p* = 0.010). Vitamin B9 levels were positively correlated with IBDQ in all patients enrolled (*r* = 0.276, *p* = 0.019).

Compared to patients with serum vitamin B9 ≥ 3 ng/mL, significantly higher levels of CRP (9.3 ± 7.3 vs. 3.9 ± 5.1 mg/L, *p* = 0.006) were found in the vitamin B9 deficient participants (Table S3).

With regard to vitamin dietary intake (Table 3), vitamin B9 consumption was significantly greater in participants with serum vitamin B9 ≥ 3 ng/mL compared to those with vitamin B9 deficiency, either expressed as total, or DFE intake (*p* = 0.038 and *p* = 0.015, respectively). Serum vitamin B9 was positively correlated with vitamin B9 total dietary intake (*r* = 0.263, *p* = 0.026) and DFE intake (*r* = 0.340, *p* = 0.004) in all patients enrolled.

#### 3.1.3. Serum Vitamin B12

In the overall study population, mean serum vitamin B12 was 447.1 ± 244.1 pg/mL (Table 2). Positive correlations of serum vitamin B12 with TBIL (*r* = 0.298, *p* = 0.007), DBIL (*r* = 0.311, *p* = 0.005) or ALB (*r* = 0.233, *p* = 0.047) were detected. In addition, serum IL-6 and serum oxidisability (lag time) were found to be negatively correlated with serum vitamin B12 (IL-6: *r* = −0.246, *p* = 0.040 and Lag time: *r* = −0.259, *p* = 0.048, respectively).

#### 3.1.4. Linear Regression Analysis

Significant positive linear associations of serum 25(OH)D with serum Fe (beta = 0.083, *p* = 0.005), as well as serum vitamin B12 with TBIL (beta = 0.357, *p* = 0.020) and DBIL (beta = 0.727, *p* = 0.033) were evident when model 2 entered the regression analysis (Table 4). Adjusting for age, sex and BMI (model 1), the following significant associations were detected: (a) serum 25(OH)D with DFE intake (beta = 0.008, *p* = 0.040), (b) serum vitamin B9 with DFE intake (beta = 0.000, *p* = 0.016), IBDQ (beta = 0.002, *p* = 0.032) or HBI (beta = −0.037, *p* = 0.001) and (c) serum vitamin B12 with DBIL (beta = 0.723, *p* = 0.015). However, these linear relationships were no longer present when model 2 was applied.

### 3.2. Analyses in CD and UC Patients Separately

Patient characteristics and serum vitamins of CD and UC patients are shown in Table 1 and Table 2, respectively. Supplementary Table S4 (Supplementary Material) presents levels of biochemical, inflammatory and oxidative stress biomarkers based on IBD type.

In regards to CD patients (*N* = 54), a statistically significant difference was detected in defensin concentration between patients of serum 25(OH)D ≥ 20 ng/mL and patients with vitamin D deficiency (8.9 ± 7.8 vs. 33.6 ± 29.4 ng/g, respectively, *p* = 0.031). Correlation analysis also revealed that vitamin D status was negatively correlated with stool defensin (*r* = −0.543, *p* = 0.013) and positively correlated with serum Fe (*r* = 0.352, *p* = 0.021) in CD patients. Vitamin B9 deficient CD patients exhibited greater HBI and CRP levels compared to those with serum vitamin B9 ≥ 3 ng/mL (HBI: 6.6 ± 3.1 vs. 3.4 ± 2.5, respectively, *p* = 0.003 and CRP: 12.4 ± 6.7 vs. 4.5 ± 5.3 mg/L, respectively, *p* < 0.001). The IBDQ score, vitamin B9 total dietary intake and DFE intake were lower in the vitamin B9 deficient compared to the non-deficient group (IBDQ: 144.7 ± 28.2 vs. 167.0 ± 29.3, respectively, *p* = 0.033; total B9 intake: 209.3 ± 71.6 vs. 326.4 ± 173.3 µg, *p* = 0.026; DFE intake: 242.0 ± 89.0 vs. 391.8 ± 217.6 µg, *p* = 0.002). According to correlation analysis in CD patients, vitamin B9 status was negatively correlated with HBI (*r* = −0.481, *p* = 0.001), serum IL-6 (*r* = −0.359, *p* = 0.015) and stool lysozyme (*r* = −0.362, *p* = 0.017). On the contrary, positive correlations were observed for serum vitamin B9 with TBIL (*r* = 0.353, *p* = 0.017), vitamin B9 total dietary intake (*r* = 0.398, *p* = 0.007) and DFE intake (*r* = 0.426, *p* = 0.004). Additionally, in CD patients, serum vitamin B12 was positively correlated with DBIL (*r* = 0.290, *p* = 0.037), while negative correlation with faecal lactoferrin was found (*r* = −0.294, *p* = 0.050).

In regards to UC (*N* = 33) patients, serum 25(OH)D levels were positively correlated with DFE intake (*r* = 0.480, *p* = 0.011). No other significant correlations were found.

Regression analysis was also conducted for CD and UC patients separately. In CD, significant linear associations of (i) serum 25(OH)D with serum Fe (beta = 0.072, *p* = 0.023) and (ii) serum vitamin B9 with vitamin B9 total dietary intake (beta = 0.000, *p* = 0.040), DFE intake (beta = 0.000, *p* = 0.040), TBIL (beta = 0.400, *p* = 0.035), urea (beta = 0.014, *p* = 0.022), HBI (beta = −0.037, *p* = 0.001) or serum IL-6 (beta = −0.009, *p* = 0.006) were evident after controlling for age, sex and BMI (model 1). When model 2 was applied, serum vitamin B9 was negatively associated with stool lysozyme (beta = −0.009, *p* = 0.020). In UC, no significant associations were found.

## 4. Discussion

In the present cross-sectional study, serum levels of vitamins B9, B12 and 25(OH)D were evaluated in 87 adult patients with IBD based on the respective serum cut off values for vitamin deficiencies [9,10]. Potential associations with disease-related biomarkers and nutritional intake were investigated. Nutrient deficiencies are likewise common in IBD, both in CD and in UC [7]. Therefore, in the present study, analysis and interpretation of data were conducted in the overall study population of IBD patients, as well as in CD and UC patients separately.

The most stable metabolite of vitamin D in serum is 25(OH)D [27] and its measurement is recommended for assessing individuals’ vitamin status [9]. Deficiency of vitamin D in IBD patients versus healthy controls is well-reported [4]. However, the presence of several factors such as impaired nutrient absorption, dietary habits, micronutrient supplementation, small bowel resection, geographical region, climate state, sun exposure, skin pigmentation and race, could explain the high variation observed in deficiency prevalence [28,29,30,31]. In our study, conducted in Attica-Greece, the results showed that vitamin D deficiency (defined as serum 25(OH)D < 20 ng/mL) was present in nearly 37% of IBD patients, an outcome that is in accordance with previously published data [4]. Deficiency of vitamin D is considered as a potential risk factor for IBD development, and vitamin D status may further affect disease activity [32]. It seems that vitamin D acts on gut epithelial permeability, physical barrier function, and T cell recruitment [33,34], while low vitamin D status is associated with increased odds of disease activity [35]. Similarly, the results of our study showed a significant negative correlation between CD disease activity (assessed by HBI) and serum 25(OH)D. The lack of correlation between UC disease activity (assessed by PMS) and serum 25(OH)D may be due to the smaller sample size compared to CD patients (33 patients with ulcerative colitis versus 54 patients with Crohn’s disease). Additionally, no significant association between vitamin D status and vitamin D dietary intake was observed in our study population. This is expected—in most populations around the world, cutaneous synthesis of vitamin D during sun exposure is the main source of vitamin D and dietary intake has minimal effect on vitamin D status.

According to recent reports, up to 45% of IBD patients are diagnosed with iron deficiency [36]. Two potential underlying mechanisms have been proposed: (a) iron loss in the stool caused by mucosal bleeding, and (b) impaired iron absorption in the bowel, due to inflammation-induced alterations in hepcidin, the key regulatory hormone of iron [37,38]. Data from 112 IBD patients with ileostomy [39] showed that vitamin D deficiency was associated with low haemoglobin (OR: 1.32, 95% CI: 1.08–1.60, *p*= 0.006). In paediatric IBD patients [40], low serum levels of 25(OH)D were significantly associated with increased hepcidin levels (beta = 0.6, *p* = 0.01) and decreased haemoglobin (beta = −0.9, *p* = 0.046). Hence, an unexpected finding of our study was the significant positive association of serum Fe with serum 25(OH)D present in the overall IBD patients adjusting for age, sex, BMI, disease, disease activity, disease duration, smoking, nutritional intake and season of recruitment. After performing regression analysis based on IBD type, this association remained significant in the CD, but not in the UC patients. The sample size did not allow us to perform further analysis based on the CD inflammation site (i.e., colonic, ileal-colonic, ileal, left-sided, other). These observations, however, point to the complementary role of serum 25(OH)D as a biomarker in assessing IBD disease severity [41].

The role of vitamin B9 is also more profound than just an anaemia biomarker. There is growing evidence that vitamin B9 has an active role in immunoregulation [42], while vitamin B9 deficiency is considered as a potent trigger of the inflammatory cascade [43]. Therefore, important finding of the present study was the significant negative association found between serum vitamin B9 and stool lysozyme in CD patients.

The reduced intake of vitamin B9-containing foods is considered as one underlying mechanism of vitamin B9 deficiency [44]. In the present study, vitamin B9 consumption was significantly greater in IBD participants with serum vitamin B9 ≥ 3 ng/mL compared to those with vitamin B9 deficiency. Furthermore, a positive association of serum vitamin B9 with DFE intake was detected, confirming the observation of enhanced vitamin B9 absorption from fortified foods [45]. Interestingly, DFE intake was positively associated with serum 25(OH)D. One plausible explanation could be that food products with added vitamin D are also fortified with folic acid, such as fortified cereals.

We also showed that CD patients with serum vitamin B9 ≥ 3 ng/mL demonstrated lower disease activity and significantly better quality of life compared to patients with deficiency, reinforcing the conclusion of Piovani and co-authors [46] that maintaining adequate serum vitamin B9 is one environmental protective factor characterised by moderate/high epidemiologic evidence.

Along with vitamin B9, vitamin B12 is also necessary for the proliferation and differentiation of erythroblasts. In IBD, data on prevalence of vitamin B12 deficiency are rather ambiguous [47,48]. Nevertheless, vitamin B12 deficiency appears to be more common in patients with ileal CD or ileal resection (7). In our study, in which patients with ileal resection were excluded, B12 deficiency (defined as <200 pg/mL) occurred in 5.7% of IBD patients of which 80.0% were diagnosed with ileal CD. Interestingly, serum B12 was positively correlated with serum albumin, an observation probably attributed to homocysteine metabolism [49]. Homocysteine might also be responsible for hepatic dysfunction. It has been reported that IBD patients with high homocysteine may often present morphological and functional disorders of the liver having normal or high serum concentrations of vitamin B12, rather than increased body stores of vitamin B12 [50]. This could explain the positive linear association observed between serum B12 and bilirubin in our study population. Interestingly, we also observed a negative correlation between serum vitamin B12 with serum IL-6 and serum oxidisability in the overall IBD patients, as well as a negative correlation with stool lactoferrin in CD patients. These results probably indicate a protective role of vitamin B12 against inflammation [49].

We recognise that sample size may limit the power of the present study. However, patients were enrolled consecutively and strict inclusion/exclusion were applied to avoid errors and bias in the results. It is worth mentioning, that core findings of the present study were in agreement with those of larger scale observational and retrospective studies, thus contributing to the validity of our results. Another limitation could be the use of subjective dietary information derived from the 24-h recall record; a diet recall cannot be considered as proof of an actual causal relation between dietary intake and vitamin deficiency. Nevertheless, our dieticians were properly trained and were able to identify unclear descriptions and ask patients to clarify them. We also recognise that red blood cell folate is considered as an indicator of longer-term vitamin B9 status compared to serum vitamin B9 levels [11]. However, almost all studies to date have used serum folate level rather than red blood cell folate, allowing the results of our study being comparable to those of previous published work. An additional limitation was that measurements of 25(OH)D were not standardised to the gold-standard method or to NIST values, so caution is required when interpreting the concentration values as they are assay-dependent.

To sum up, vitamin B9 deficient patients demonstrated higher CRP levels compared to patients with serum vitamin B9 ≥ 3 ng/mL, verifying the results of in vitro and animal studies regarding the anti-inflammatory properties of vitamin B9 in IBD. When tested in CD patients separately, vitamin B9 was negatively associated with stool lysozyme. These results are in agreement with a higher vitamin B9 dietary intake (both total and DFE) observed in the vitamin B9 non-deficient versus the deficient patients (both IBD and CD alone), highlighting the necessity of adequate vitamin B9 consumption from the daily diet. We also observed a negative correlation between serum vitamin B12 with serum IL-6 and serum oxidisability in the overall IBD patients, as well as a negative correlation with stool lactoferrin in CD patients. These results probably indicate the anti-inflammatory and the antioxidant properties of vitamin B12 in IBD. Moreover, a significant positive association of serum Fe with serum 25(OH)D was found, confirming the protective role of vitamin D in iron deficiency.

## 5. Conclusions

In conclusion, deficiencies of vitamins D, B9 and B12 were evident in IBD patients. Significant associations between these serum vitamins and clinical, biochemical and oxidative stress markers were detected, thus confirming previous scientific publications of the pivotal role of vitamins in IBD severity and prognosis.

This is the first study elucidating the association of IBD-related indices with the status of three major vitamins, i.e., vitamin D, B9 and B12, in a Greek population of IBD patients. The serum vitamin profile may be a complementary biomarker for the evaluation of disease activity next to serum and stool inflammatory biomarkers. Nevertheless, larger observational cohort studies to assess all these IBD characteristics in regards to serum vitamins are required.

## Figures and Tables

**Table 1:** Demographic and clinical characteristics of all inflammatory bowel disease (IBD), Crohn’s disease (CD) and ulcerative colitis (UC) patients.

	All (*N* = 87)	CD (*N* = 54)	UC (*N* = 33)	*p*
Sex (*N*)	46 F/41 M	26 F/28 M	15 F/18 M	
Age (mean ± SD)	39.6 ± 13.1 years	39.1 ± 13.5 years	40.3 ± 12.4 years	0.64
Smoking (N)	55 NO/31 YES/1 NA	19 NO/35 YES	20 NO/12 YES/1 NA	
BMI (mean ± SD)	24.6 ± 4.9 kg/m^2^	24.8 ± 5.2 kg/m^2^	24.4 ± 4.3 kg/m^2^	0.79
Disease duration (mean ± SD)	9.3 ± 7.0 years	9.5 ± 7.1 years	9.1 ± 6.8 years	0.87
Disease location (*N*)				
Colonic	5 (5.8%)	3 (5.5%)	2 (6.1%)	
Ileal-colonic	19 (21.8%)	19 (35.2%)	0 (0.0%)	
Ileal	16 (18.4%)	16 (29.6%)	0 (0.0%)	
Left-sided	8 (9.2%)	1 (1.9%)	7 (21.2%)	
Pancolitis	20 (23.0%)	0 (0.0%)	20 (60.6%)	
Other	19 (21.8%)	15 (27.8%)	4 (12.1%)	
Disease activity (*N*)				
Relapse	38 (43.7%)	21 (38.9%)	17 (51.5%)	
Remission	49 (56.3%)	33 (61.1%)	16 (48.5%)	
HBI	4.1 ± 2.8	4.1 ± 2.8	-	
PMS	2.5 ± 2.1	-	2.5 ± 2.1	
IBDQ score	162.0 ± 30.7	161.0 ± 30.3	163.7 ± 31.9	0.56

Data are mean values ± standard deviation (SD). IBD, inflammatory bowel disease; CD, Crohn’s disease; UC, ulcerative colitis; F, female; M, male; NA, non-available; BMI, body mass index; IBDQ, Inflammatory Bowel Disease Questionnaire; HBI, Harvey Bradshaw Index; PMS, Partial Mayo Score. *P*: differences between CD and UC patients were analysed using Student’s *t*-test for normally distributed variables or Mann–Whitney U test for those not normally distributed. Difference was considered significant at *p* < 0.05.

**Table 2:** Serum vitamin status of all IBD, CD and UC patients.

Serum Vitamins	All (*N* = 87)	CD (*N* = 54)	UC (*N* = 33)	*p*
25(OH)D (ng/mL) (*N* = 74)	20.7 ± 7.5	20.4 ± 6.8	20.8 ± 7.7	0.79
<20 ng/mL (*N* = 32)	14.2 ± 3.1	14.7 ± 3.2	13.1 ± 2.9	0.16
≥20 ng/mL (*N* = 42)	25.5 ± 5.2	25.4 ± 4.9	25.5 ± 5.6	0.93
Vitamin B9 (ng/mL) (*N* = 74)	5.3 ± 2.7	5.3 ± 2.5	5.8 ± 2.9	0.54
<3 ng/mL (*N* = 16)	2.5 ± 0.4	2.4 ± 0.5	2.5 ± 0.2	0.91
≥3 ng/mL (*N* = 58)	6.3 ± 2.4	6.2 ± 2.2	6.5 ± 2.8	0.80
Vitamin B12 (pg/mL) (*N* = 87)	447.1 ± 244.1	450.4 ± 286.4	430.3 ± 113.4	0.25
<200 pg/mL (*N* = 5)	155.2 ± 18.6	160.8 ± 16.0	133.0	-
≥200 pg/mL (*N* = 82)	460.3 ± 231.2	473.6 ± 285.2	439.6 ± 101.7	0.37

Data are mean values ± standard deviation (SD). IBD, inflammatory bowel disease; CD, Crohn’s disease; UC, ulcerative colitis; *P*: differences between CD and UC patients were analysed using Student’s *t*-test for normally distributed variables or Mann–Whitney U test for those not normally distributed. Difference was considered significant at *p <* 0.05.

**Table 3:** Dietary intake of vitamins in all enrolled IBD patients with or without vitamin deficiencies.

Dietary Intake	25(OH)D < 20 ng/mL (*N* = 32)	25(OH)D ≥ 20 ng/mL (*N* = 42)	*p*	Vitamin B9 < 3 ng/mL (*N* = 16)	Vitamin B9 ≥ 3 ng/mL (*N* = 58)	*p*
Vitamin D (µg)	1.4 ± 1.2	1.5 ± 1.4	0.89	1.4 ± 1.2	1.5 ± 1.4	0.95
Vitamin B9 total (µg)	310.7 ± 173.4	351.1 ± 209.5	0.50	250.2 ± 198.0	305.1 ± 152.5	0.038
DFE intake (µg)	313.3 ± 155.8	424.9 ± 284.1	0.88	235.8 ± 90.1	358.5 ± 192.2	0.015
Vitamin B12 (µg)	4.2 ± 4.6	4.0 ± 4.5	0.78	4.1 ± 4.5	3.9 ± 4.1	0.37

Data are mean values ± standard deviation (SD). IBD, inflammatory bowel disease; DFE, dietary folate equivalent. *p*: differences between two independent groups were analysed using Student’s *t*-test for normally distributed variables or Mann–Whitney U test for those not normally distributed. Difference was considered significant at *p <* 0.05; significant *p* values are marked bold in the table.

**Table 4:** Regression analysis addressing the associations between vitamins and data showing significant correlations in overall IBD patients.

Serum Vitamins	Unadjusted		Model 1		Model 2	
	Beta	*p*	Beta	*p*	Beta	*p*
25(OH)D (ng/mL)						
DFE dietary intake (µg)	0.008	**0.033**	0.008	**0.040**	0.005	0.45
Serum Fe (μg/dL)	0.070	**0.010**	0.058	**0.032**	0.083	**0.005**
Vitamin B9 (ng/mL)						
Vitamin B9 total dietary intake (µg)	0.000	0.16	0.000	0.20	0.000	0.67
DFE dietary intake (µg)	0.000	**0.018**	0.000	**0.016**	0.000	0.59
Serum IL-6 (pg/mL)	−0.006	**0.027**	−0.006	0.052	−0.004	0.23
IBDQ	0.002	**0.020**	0.002	**0.032**	0.000	0.73
HBI	−0.034	**0.002**	−0.037	**0.001**	−0.028	0.17
Total antioxidant capacity (lag time, sec)	−5.533 × 10^−5^	0.06	−5.066 × 10^−5^	0.09	1.100 × 10^−5^	0.75
Vitamin B12 (pg/mL)						
Serum TBIL (mg/dL)	0.273	**0.033**	0.317	**0.018**	0.357	**0.020**
Serum DBIL (mg/dL)	0.558	**0.039**	0.723	**0.015**	0.727	**0.033**
Serum albumin (g/dL)	0.015	**0.039**	0.012	0.11	0.011	0.16

IBD, inflammatory bowel disease; DFE, dietary folate equivalent; Fe, iron; IL-6, interleukin-6; IBDQ, Inflammatory Bowel Disease Questionnaire; HBI, Harvey-Bradshaw Index; TBIL, total bilirubin; DBIL, direct bilirubin. Significance level was set at *p <* 0.05. Model 1: adjusted for age, sex and BMI. Model 2: adjusted for age, sex, BMI, disease, disease activity, disease duration, smoking, nutritional intake and season of recruitment; significant *p* values are marked bold in the table.

## Supplementary Materials

The following are available online at https://www.mdpi.com/2072-6643/12/12/3734/s1, Table S1. Biochemical profile results according to vitamin status in overall IBD patients. Table S2. Stool inflammatory biomarkers according to vitamin status in overall IBD patients. Table S3. Inflammatory and oxidative stress biomarkers in serum or plasma according to vitamin status in overall IBD patients. Table S4. Inflammatory and oxidative stress biomarkers in all IBD, CD and UC patients.
