# Supplementary material for: Serum Vitamins D, B9 and B12 in Greek Patients with Inflammatory Bowel Diseases
Source: Nutrients. 2020 Dec 4;12(12):3734. doi: 10.3390/nu12123734 (PMC7761805; doi:10.3390/nu12123734)
Supplement: Supplementary file 1 [file nutrients-12-03734-s001.pdf]

Supplementary Material

Serum vitamins D, B9 and B12 in Greek patients with Inflammatory Bowel Diseases

Supplementary Table S1. Biochemical profile results according to vitamin status in overall IBD patients

**Markers**	**25(OH)D <20 ng/mL (N=32)**	**25(OH)D ≥20 ng/mL (N=42)**	***P***	**Vitamin B9 <3 ng/mL (N=16)**	**Vitamin B9 ≥3 ng/mL (N=58)**	***P***
Amylase (IU/L)	62.9±22.4	70.8±29.4	0.14	63.0±26.4	63.3±18.2	0.92
Fibrinogen (mg/dL)	272.7±56.5	254.0±62.7	0.08	285.4±49.1	262.6±58.2	0.18
Fe (μg/dL)	53.7±30.6	68.2±30.4	**0.048**	49.5±29.8	63.6±27.3	0.06
TBIL (mg/dL)	0.3±0.2	0.4±0.2	0.08	0.3±0.2	0.3±0.2	0.14
DBIL (mg/dL)	0.2±0.1	0.2±0.1	0.30	0.1±0.1	0.2±0.1	0.26
GLU (mg/dL)	85.6±13.6	85.5±15.6	0.99	89.9±13.3	86.7±17.7	0.25
Urea (mg/dL)	28.3±6.7	32.0±5.5	**0.016**	27.4±6.2	31.1±5.7	**0.028**
TC (mg/dL)	182.1±49.5	169.7±45.8	0.06	155.0±40.2	176.1±43.7	0.11
HDL-C (mg/dL)	55.4±17.1	53.0±20.5	0.60	46.3±9.6	52.5±18.5	0.07
LDL-C (mg/dL)	106.4±38.3	92.1±36.4	0.11	86.4±31.1	100.9±37.2	0.16
TG (mg/dL)	101.5±47.0	122.5±57.5	0.06	114.4±68.5	113.5±55.8	0.67
AST (IU/L)	15.1±4.7	17.0±6.6	0.16	15.0±7.0	15.8±4.6	0.70
ALT (IU/L)	17.1±9.0	17.9±9.5	0.72	17.1±9.8	16.8±7.0	0.84
γ-GT (IU/L)	17.7±10.7	16.7±7.5	0.43	15.8±6.8	18.0±9.9	0.51

ALP (IU/L)	63.5±17.0	65.8±20.3	0.57	62.9±14.2	64.5±20.4	0.78
LDH (U/L)	150.8±36.7	148.7±30.7	0.80	137.4±34.2	152.2±35.5	0.15

Data are mean values ± standard deviation (SD). IBD, inflammatory bowel disease; Fe, iron; TBIL, total bilirubin; DBIL, direct bilirubin; GLU, glucose; TC, total cholesterol; HDL-C, high-density lipoprotein cholesterol; LDL-C, low-density lipoprotein cholesterol; TG, triglycerides; AST, aspartate aminotransferase; ALT, alanine aminotransferase; γGT, γ-glutamyl transpeptidase; ALP, alkaline phosphatase; LDH, lactate dehydrogenase. *P*: differences between two independent groups were analysed using Student's t-test for normally distributed variables or Mann-Whitney U test for those not normally distributed. Difference was considered significant at $P<0.05$.

Supplementary Table S2. Stool inflammatory biomarkers according to vitamin status in overall IBD patients

**Stool biomarkers**	**25(OH)D <20 ng/mL (N=32)**	**25(OH)D ≥20 ng/mL (N=42)**	***P***	**Vitamin B9 <3 ng/mL (N=16)**	**Vitamin B9 ≥3 ng/mL (N=58)**	***P***
Calprotectin (μg/g)	1005.1±1015.7	1066.4±1501.6	0.85	1176.7±1254.3	967.2±1142.6	0.57
Defensin (ng/g)	23.4±27.3	18.7±25.6	0.60	24.9±36.8	15.4±17.7	0.31
Lysozyme (μg/g)	12.0±11.3	10.5±7.4	0.59	13.3±9.0	11.8±11.1	0.18
Lactoferrin (μg/g)	55.8±59.9	76.8±109.7	0.38	98.9±127.7	63.2±92.4	0.93

Data are mean values ± standard deviation (SD). IBD, inflammatory bower disease. *P*: differences between two independent groups were analysed using Student's t-test for normally distributed variables or Mann-Whitney U test for those not normally distributed. Difference was considered significant at $P<0.05$.

Supplementary Table S3. Inflammatory and oxidative stress biomarkers in serum or plasma according to vitamin status in overall IBD patients

**Biomarkers**	**25(OH)D <20 ng/mL (N=32)**	**25(OH)D ≥20 ng/mL (N=42)**	***P***	**Vitamin B9 <3 ng/mL (N=16)**	**Vitamin B9 ≥3 ng/mL (N=58)**	***P***
**Inflammation**						
CRP (mg/L)	5.6±7.8	3.8±5.2	0.40	9.3±7.3	3.9±5.1	**0.006**
IL-6 (pg/mL)	8.1±8.0	8.5±11.1	0.36	11.5±12.6	7.4±8.7	0.12
IL-10 (pg/mL)	5.3±1.4	5.6±2.2	0.46	5.1±1.5	5.4±1.8	0.70
IL-17 (pg/mL)	29.6±19.6	23.5±13.9	0.10	18.7±9.8	23.4±14.5	0.31
IL-22 (pg/mL)	24.1±25.2	22.1±20.5	0.48	13.2±12.8	23.5±23.9	0.28
IL-11 (pg/mL)	218.2±159.6	284.6±260.4	0.89	149.7±36.1	282.3±242.6	0.68
**Oxidative stress**						
Uric acid (mg/dL)	4.3±1.4	4.9±1.4	0.21	5.2±1.5	4.6±1.3	0.16
Albumin (g/dL)	43.0±3.3	43.7±3.0	0.55	42.2±3.8	43.2±3.2	0.33
Cysteine (nmol/L)	27.1±2.2	27.3±4.8	0.46	28.3±4.0	27.7±3.8	0.57
Total antioxidant capacity (lag time, sec)	2754.5±953.7	2706.8±1066.6	0.89	3078.0±1301.2	2641.3±875.1	0.27
MPO (ng/mL)	67.4±55.7	84.3±64.7	0.26	70.9±55.0	87.3±69.3	0.67

Data are mean values ± standard deviation (SD). IBD, inflammatory bower disease; CRP, C-reactive protein; IL-, interleukin; MPO, myeloperoxidase. *P*: differences between two independent groups were analysed using Student's t-test for normally distributed variables or Mann-Whitney U test for those not normally distributed. Difference was considered significant at *P<0.05*.

Supplementary Table S4. Inflammatory and oxidative stress biomarkers in all IBD, CD and UC patients

**Bowel inflammatory markers**	**All (N=87)**	**CD (N=54)**	**UC (N=33)**	***P***
Calprotectin (μg/g)	1084.4±1344.0	1005.1±1125.2	1216.6±1654.2	0.90
Defensin (ng/g)	19.1±24.2	18.4±22.2	19.9±27.4	0.80
Lysozyme (μg/g)	11.5±10.1	11.4±8.5	11.7±12.7	0.33
Lactoferrin (μg/g)	69.3±95.5	56.6±89.9	93.1±102.9	**0.052**
**Serum Inflammatory markers**				
CRP (mg/L)	4.6±5.8	5.8±6.4	2.9±4.2	0.051
IL-6 (pg/mL)	7.7±9.3	8.5±9.8	6.4±8.5	0.31
IL-10 (pg/mL)	5.5±2.0	5.3±1.5	6.0±2.5	0.29
IL-17 (pg/mL)	24.4±16.1	24.9±17.4	23.6±13.9	0.96
IL-22 (pg/mL)	22.2±21.5	21.2±21.5	24.4±22.2	0.56
IL-11 (pg/mL)	262.7±217.8	264.6±203.4	259.7±250.0	0.81
**Serum ox. damage/antioxidant markers**				
Uric acid (mg/dL)	4.7±1.4	5.0±1.4	4.1±1.2	**0.005**
Albumin (g/dL)	43.1±3.2	42.7±3.2	43.7±3.2	0.09
Cysteine (nmol/L)	27.4±3.7	27.7±3.7	27.0±3.8	0.28
Total antioxidant capacity (lag time, sec)	2724.6±1022.4	2656.3±921.7	2850.3±1196.1	0.63
MPO (ng/mL)	81.5±65.2	78.7±66.2	85.5±64.4	0.49
**Biochemical indices**				
Amylase (IU/L)	66.3±21.4	66.9±20.0	65.3±23.9	0.74
Fibrinogen (mg/dL)	264.2±59.6	266.1±65.3	261.2±50.3	0.73
Fe (μg/dL)	61.3±30.5	59.8±31.2	63.8±29.7	0.54
TBIL (mg/dL)	0.3±0.2	0.3±0.2	0.3±0.1	0.96
DBIL (mg/dL)	0.2±0.1	0.2±0.1	0.1±0.1	0.83
GLU (mg/dL)	85.8±14.5	87.0±14.3	83.7±14.9	0.33
Urea (mg/dL)	30.2±6.2	29.7±6.0	31.1±6.4	0.33
TC (mg/dL)	169.9±40.7	163.9±42.1	179.4±37.2	0.10
HDL-C (mg/dL)	53.1±18.8	50.5±14.1	57.3±24.3	0.16
LDL-C (mg/dL)	99.3±37.9	96.4±42.9	104.1±29.7	0.33
TG (mg/dL)	115.0±58.8	121.3±61.1	104.8±54.3	0.19

AST (IU/L)	15.6±5.0	15.6±5.5	15.5±4.3	0.90
ALT (IU/L)	16.5±7.6	17.2±8.3	15.4±6.4	0.53
γ-GT (IU/L)	17.1±9.1	18.0±9.2	15.8±8.9	0.21
ALP (IU/L)	64.2±18.9	63.8±18.6	64.9±19.6	0.80
LDH (U/L)	148.9±34.3	146.6±34.2	152.6±34.7	0.44

Data are mean values ± standard deviation (SD). IBD, inflammatory bowel disease; CD, Crohn's disease; UC, ulcerative colitis; CRP, C-reactive protein; IL-, interleukin; MPO, myeloperoxidase; Fe, iron; TBIL, total bilirubin; DBIL, direct bilirubin; GLU, glucose; TC, total cholesterol; HDL-C, high-density lipoprotein cholesterol; LDL-C, low-density lipoprotein cholesterol; TG, triglycerides; AST, aspartate aminotransferase; ALT, alanine aminotransferase; γGT, γ-glutamyl transpeptidase; ALP, alkaline phosphatase; LDH, lactate dehydrogenase. *P*: difference between CD and UC patients were analysed using Student's t-test for normally distributed variables or Mann-Whitney U test for those not normally distributed. Difference was considered significant at $P<0.05$.
